# Genetic analysis and clinical assessment of four patients with Glycogen Storage Disease Type IIIa in China

**DOI:** 10.1186/s12881-018-0560-6

**Published:** 2018-04-04

**Authors:** Yu Zhang, Mingming Xu, Xiaoxia Chen, Aijuan Yan, Guoyong Zhang, Zhenguo Liu, Wenjuan Qiu

**Affiliations:** 1 Department of Neurology, Xinhua Hospital Affiliated to Shanghai Jiao Tong University School of Medicine, 1665 Kong jiang Road, Shanghai, 200092 People’s Republic of China; 2 Department of Pediatric Endocrinology/Genetics, Shanghai Institute for Pediatric Research, Xinhua Hospital Affiliated to Shanghai Jiao Tong University School of Medicine, Shanghai, 200092 China

**Keywords:** Glycogen storage disease IIIa, *AGL* gene, Clinical characteristics, Chinese

## Abstract

**Background:**

Glycogen Storage Disease Type III (GSD III) is a rare autosomal recessive metabolic disorder caused by *AGL* gene mutation. There is significant heterogeneity between the clinical manifestations and the gene mutation of *AGL* among different ethnic groups. However, GSD III is rarely reported in Chinese population.

**Case presentation:**

In this study, we aimed to study the genetic and clinical characteristics of four patients with GSD IIIa from China, especially the neurological manifestations. Meanwhile, we conducted a literature review of GSD IIIa cases reported in Chinese population to investigate the relationship between genotype and phenotype.

**Conclusions:**

Three different *AGL* gene mutations were identified in our patients: c.206dupA, c.1735 + 1G > T and c.2590 C>T. Moreover, progressive myopathy accompanied by elevated creatine kinase level was the main manifestation of our patients in adolescents. Our results showed that *AGL* c.206dupA was a novel mutation and caused severe clinical manifestations. *AGL* c.1735 + 1G > T might be a recurrent mutation in the Chinese population. Genetic analysis of *AGL* gene mutation combined with muscle magnetic resonance imaging (MRI) might provide greater benefit to the patient in diagnosing GSD IIIa, rather than an invasive diagnostic procedure of biopsy.

**Electronic supplementary material:**

The online version of this article (10.1186/s12881-018-0560-6) contains supplementary material, which is available to authorized users.

## Background

Glycogen Storage Disease Type III (GSD III) is a rare autosomal recessive metabolic disorder. There are two major clinical types of GSD III. GSD IIIa involves liver and muscle while GSD IIIb only affects the liver. GSD III commonly presents with growth retardation, fasting hypoglycemia, hepatomegaly, and seizures during childhood. The neuromuscular manifestations of GSD IIIa are mainly reported in adults [1–3]. GSD III is caused by *AGL* gene mutation leading a deficiency of glycogen debrancher enzyme activity. The human *AGL* gene is located on chromosome 1p21 and consists of 35 exons spanning ~ 85 kb of genomic DNA. *AGL* gene is expressed from the third exon [4]. Genetic analysis of the *AGL* gene in several ethnic populations has revealed over 150 different *AGL* gene mutations [5]. However, few *AGL* mutations have been reported in the Chinese population in mainland China. In this study, we reported four Chinese patients with GSD IIIa and analyzed the relationship between genotype and phenotype of GSD IIIa in the Chinese population.

## Case presentation

Our study included three male and one female GSD IIIa Chinese patients from three families. Patients 3 and 4 were siblings from one family. When these patients were admitted to our hospital, their clinical information, such as age, gender, height, weight, liver function and clinical symptoms were collected. Then, routine laboratory test, echocardiography, electromyogram, abdominal ultrasound, muscle MRI and neuropsychological test were conducted. All of this clinical information of the first visit and two years follow-up were shown in Table 1. The guideline for the diagnosis of GSD III had been followed [5].

Our patients mainly complained physical weakness accompanied by elevated creatine kinase level. Although patients had not received high-protein and uncooked cornstarch (UCS) diet therapy in childhood, growth retardation was not observed after adolescence. Treatment with high-protein and UCS diet was implemented in all patients after the first visit, according to preprandial blood glucose and the level of abnormal alanine aminotransferase (ALT) and aspartate aminotransferase (AST). After high-protein and UCS diet therapy, clinical manifestations of weakness of our patients were significantly relieved at the last follow-up. Currently, their therapy followed standard treatment guidelines, with UCS in 1.5 g/kg/time for 1–3 times per day and protein in 2 g/kg/d.

The results of laboratory tests showed the level of creatine kinase (CK) was significantly increased among all patients, but ketonic hypoglycemia was only detected in patient 1. Serum cholesterol and triglyceride were normal in patient 1 but they were elevated in other patients. After a high-protein and UCS diet therapy for two years, the level of CK was still 5–10 times over the upper limit of normal, but preprandial blood glucose, serum cholesterol and triglyceride of all patients had nearly normalized at the last follow-up.

All our patients showed asymptomatic cardiac hypertrophy accompanied by normal ejection fraction and cardiac conduction (Table 1). Meanwhile, hepatomegaly accompanied by abnormal ALT and AST was common during our patients’ childhood. After a high-protein and UCS diet therapy for two years, cardiac hypertrophy of our patients did not deteriorated. Moreover, levels of AST and ALT had decreased and hepatic adenoma was not detected in our patients. We didn’t observe hepatic cirrhosis or hepatocellular carcinoma in our patients by liver ultrasound or laboratory tests. Indeed, we did not perform the liver biopsy which was invasive and potentially detrimental to the patients.

**Table 1:** Clinical features and gene mutation of GSD IIIa patients

	Patient 1		Patient 2		Patient 3		Patient 4	
	first visit	last follow-up	first visit	last follow-up	first visit	last follow-up	first visit	last follow-up
Gender	male		male		female		male	
Onset age (years)	1	/	2	/	1	/	1	/
Onset symptoms	abdominal distension	/	abdominal distension	/	abdominal distension	/	abdominal distension	/
Age (years)	16	18	32	34	22	24	14	16
Height(cm)	167	169	170	170	164	164	147	ND
Weight(kg)	60	62	75	75	77	80	46.5	ND
Complains	weakness, syncope, headache, irritability,	weakness relief	weakness	weakness relief	weakness	weakness relief	weakness	weakness relief
Myopathy	+	+/−	+	+/−	+	+/−	+	+/−
Hepatomegaly (below the costal margin>2 cm)	+	–	+	–	+	–	+	–
Cardiomypathy (LVPW>3.7 mm)	–	–	LVH	LVH (stationary)	LVH	LVH (stationary)	LVH	ND
Electrocardiography	–	ND	–	ND	–	ND	–	ND
Hypoglycemia	+	–	–	–	–	–	–	ND
Electromyogram	Mup in proximal limbs	ND	Mup in proximal limbs	ND	Mup in proximal limbs	ND	Mup in proximal limbs	ND
Muscle MRI	+	ND	+	ND	+	ND	+	ND
NCS	–	ND	–	ND	–	ND	–	ND
Creatine kinase (U/L)(24–195)	4000	1470	1126	2377	1190	ND	2785	ND
Alanine aminotransferase (U/L)(0–50)	199	244	103	104	78	32	108	ND
Aspartate aminotransferase (U/L)(0–50)	202	294	81	116	75	41	196	ND
Gamma-glutamyl transpeptidase(U/L)(16–73)	120	202	27	28	25	ND	70	ND
Lactate (mmol/l) (0.1–2.7)	2.5	ND	2.3	ND	1.8	ND	1.2	ND
Uric acid (mmol/l) (208–428)	346	332	359	581	293	ND	300	ND
Cholesterol (mmol/L) (3.55–5.20)	4.47	5.1	8.61	7.14	5.99	5.06	4.0	ND
Triglyceride (mmol/L) (0.45–1.81)	1.19	1.6	4.43	5.45	3.09	3.41	0.49	ND
Fasting blood glucose (mmol/L)(3.89–6.11)	2.3	3.8	5.0	4.18	4.5	6.23	4.41	ND
Urine ketone body	+	–	–	–	+	–	+	–
*AGL* mutation	*AGL* c.206dupA (homozygous)		*AGL* c.1735 + 1G > T (homozygous)		*AGL* c.1735 + 1G > T; c.2590 G>T (heterozygous)		*AGL* c.1735 + 1G > T; c.2590 G>T (heterozygous)	

*LVPW* Left Ventricular Posterior Wall, *LVH* Left ventricular hypertrophy, *MUP* Motor unit potential, *NCS* Nerve Conduction Studies, *ND* not done

**Fig. 1:**
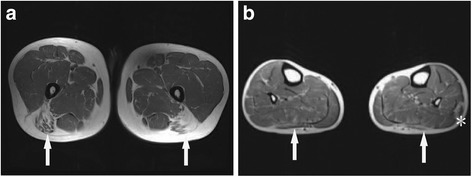
Muscle MRI comprising T1-weighted images of the thigh and lower leg from patient 2. **a**. Transverse cuts from the mid-thigh muscles showed mild increase in signal intensity within the femoris long head (arrows); **b**, Transverse cuts from the lower leg showed mild increase in signal intensity within the peroneus longus (arrows) and there was atrophy of gastrocnemius lateral heads (asterisk)

To better identify the neurological manifestations of GSD IIIa in our patients, nerve conduction studies (NCS), electromyogram (EMG), muscle MRI, brain MRI, electroencephalogram and neuropsychological tests were performed. All patients showed normal results in NCS, brain MRI and electroencephalogram, but the results of EMG were abnormal. Myopathic motor unit potential was detected in all patients, but the specificity and sensitivity were not satisfactory. Notably, Muscle MRI transverse sections from the mid-thigh and lower leg muscles of patient 2 presented mild T1 signal intensity in the long head of femoris and peroneus longus muscles (arrows), indicating an increase in adipose-like tissue (Fig. 1).What’s more, lateral heads of gastrocnemius muscles showed atrophy in patient 2 (Fig. 1). In general, the posterior and lateral muscles of the lower limbs were mostly affected (Fig. 1).

### DNA sequence analysis of *AGL* gene

The genomic DNA of all family members was extracted from peripheral blood by using the standard phenol–chloroform extraction method. Polymerase chain reaction using the primers located in the flanking introns was conducted to amplify the *AGL* gene. *AGL* gene mutations were screened by direct sequencing using an ABI Prism 3100 Genetic Analyzer (Applied Biosystems, USA). The sample sequences were compared with the genomic DNA sequence of *AGL* (GenBank accession no.NM_000642.2).

**Fig. 2:**
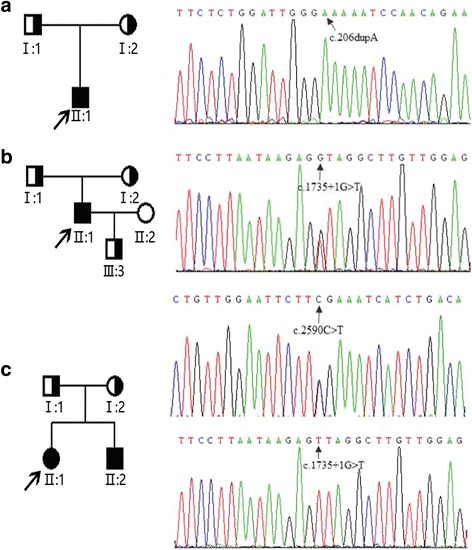
Pedigrees of our patients with GSD IIIa and mutation analysis of *AGL* gene. Filled circles and squares represent affected females and males, respectively. Proband is indicated with an arrow; **a**. patient 1 (II:1) from family 1. The DNA sequence chromatogram of the proband indicates *AGL* c.206dupA homozygous mutation indicated with an arrow; **b**. patient 2 (II:1) from family 2. The DNA sequence chromatogram of the proband indicates *AGL* c.1735 + 1G > T homozygous mutation with an arrow; **c**. patient 3 (II:1) and patient 4 (II:2) from family 3. The DNA sequence chromatogram of the proband indicates *AGL* C.1735 + 1G > T and *AGL* c.2590 C>T compound heterozygous mutations indicated with arrows

We found three different *AGL* mutations (Fig. 2) in our four patients with GSD IIIa from three families. Among these three mutations, one mutation was novel and the other two were reported. Patient 1, who still had frequent episodes of hypoglycemia in adolescence, carried a homozygous insert mutation *AGL* c.206dupA (p. N69Kfs*8). This *AGL* c.206dupA mutation is a frameshift mutation causing premature termination codons in the exon4 and is predicted to be “disease-causing mutation” by MutationTaster (http://www.mutationtaster. org/).The *AGL* c.206dupA mutation has not been documented in any public database, nor in our internal exonic database of OMIM genes from 1000 individuals. Patient 2 was homozygous for the *AGL* c.1735 + 1G > T (IVS14 + 1G > T) splice site mutation, which was predicted to impair normal splicing. For patients 1 and 2, the homozygous mutations were inherited from their parents separately. Patients 3 and 4 had compound heterozygous mutations. One of the mutations is *AGL* c.2590 C>T which is inherited from their mother. This mutation results in substitution of arginine at codon 864 by termination codon (p.R864X) and causes premature termination. Another mutation is *AGL* c.1735 + 1G > T which is inherited from their father.

### Literature review

Literature review of GSD IIIa reported in Chinese patients was conducted by searching for studies published from 1996 to 2017, with the keywords “Glycogen Storage Disease Type III”, “glycogen debranching enzyme” and “*AGL* gene”.The databases included Pub Med, Medline, VIP database and Chinese Biology Medicine. Only studies published in English or Chinese were included. All relevant papers were read carefully.

We had reviewed five papers including eighteen cases of GSD IIIa Chinese patients [6–10]. The detailed clinical information of these patients were shown in Additional file 1: Table S1. There was fifteen male and three female cases. The mean age was 5.7 ± 10.3 years old (median 4 years; range 1–46 years). Hepatomegaly and myopathy were common clinical manifestations in Chinese GSD IIIa patients on different stages of individual development, but hepatic adenoma was rarely reported. CK, ALT, AST, cholesterol, triglyceride and fasting blood-glucose were significantly abnormal in GSD IIIa patients in China. A variety of mutations of the *AGL* gene were found in Chinese GSD IIIa patients, including deletion, missense, nonsense and splicing mutations. There were five patients with *AGL* c.1735 + 1G > T mutation. Therefore, *AGL* c.1735 + 1G > T might be the most recurrent mutation in Chinese patients.

## Discussion and conclusions

GSD III is caused by mutations in the *AGL* gene. Over 150 different mutations in the *AGL* gene have been identified. However, few *AGL* mutations have been reported in mainland China [4, 5]. Genetic analysis of our four Chinese GSD IIIa patients revealed three different mutations in *AGL* gene. One novel frameshift insert mutation c.206dupA (N69Kfs*8) is located in exon4. It is likely be classified as a pathogenic mutation by following the ACMG/AMP sequence variant pathogenicity classification guideline [11]. As to the relationship between genotype and phenotype, we found the patient with *AGL* c.206dupA mutation showed severe clinical manifestations compared with the other patients. The patient carrying the *AGL* c.206dupA mutation still suffered severe hypoglycemia symptoms even in adolescence, which were absent in other patients. These manifestations indicated *AGL* c.206dupA mutation might cause more severe functional deficiency of the glycogen debranching enzyme than other mutations. Therefore, the patient with *AGL* c.206dupA mutation required a high-protein and UCS diet therapy even in adults. Then, *AGL* c.1735 + 1G > T mutation, which is first reported in Japan [12], is predicted to impair normal splicing. *AGL* c.1735 + 1G > T mutation might be a recurrent mutation in Chinese patients and should be given priority when gene detection is conducted in Chinese GSD IIIa patients. Moreover, AGL c.2590 C>T,which is first reported by Caucasians [13], is predicted to lead to premature termination, which completely abolishes the enzyme activity. In summary, the patient carrying *AGL* c.206dupA homozygous mutation suffered more severe disease, whereas patient 2 carrying the splicing homozygous mutation had milder disease. This observation is in keeping with the notion that some residual functional protein may be produced from the splicing mutation.

We have collected detailed clinical information of four Chinese GSD IIIa patients from three families. We revealed clinic characteristics of Chinese GSD IIIa: Firstly, all of our patients mainly complained of weakness when running and climbing stairs, and a high-protein and UCS diet therapy could improve the symptom of muscle weakness effectively. Secondly, our patients showed growth retardation in childhood, but they can catch up to normal level in adolescence. Mental or physical development disabilities did not appear in most of patients. Thirdly, asymptomatic hepatomegaly is common in our patients. Liver and cardiac dysfunction is not significant in all of our patients. Finally, our study showed muscle MRI combined with genetic testing might be reliable, convenient and less invasive compared with muscle biopsy [14, 15].

In conclusion, our study provides a comprehensive overview of genetic and clinical features of GSD IIIa in China. Our results revealed that muscle weakness accompanied with high level of CK are the main clinical manifestations in adolescence. Cardiomyopathy and hepatic adenoma needs long term follow-up. *AGL* c.206dupA mutation is a novel mutation leading severe clinical manifestations and *AGL* C.1735 + 1G > T might be a recurrent mutation in Chinese patients with GSD IIIa. Moreover, clinical history, muscle MRI and genetic testing may provide greater benefit to the patient in diagnosing GSD IIIa.

## Additional file

Additional file 1: **Table S1**. Clinical and genetic features of GSD IIIa patients reported in China. Literature review of GSD IIIa reported in Chinese patients was conducted by searching for studies published from 1996 to 2017. (DOCX 17 kb)

## Abbreviations

EMG: Electromyogram;
GSD III: Glycogen storage disease type III
NCS: Nerve conduction studies
UCS: Uncooked cornstarch
