# Supplementary material for: Genetic analysis and clinical assessment of four patients with Glycogen Storage Disease Type IIIa in China
Source: BMC Med Genet. 2018 Apr 4;19:54. doi: 10.1186/s12881-018-0560-6 (PMC5883582; doi:10.1186/s12881-018-0560-6)
Supplement: Supplementary file 1 — Table S1. Clinical and genetic features of GSD IIIa patients reported in China. Literature review of GSD IIIa reported in Chinese patients was conducted by searching for studies published from 1996 to 2017. (DOCX 17 kb) [file 12881_2018_560_MOESM1_ESM.docx]

**Supplementary table 1.Clinical and genetic features of GSD III patients reported in China**

| No | gender | age(years) | height(cm) | weight(kg) | *AGL* mutation | complained | myasthenia | hepatomegaly | cardiomypathy | hypoglycemia | Other feathers | CK(U/L)  (24-195) | ALT(U/L) (0-50) | AST(U/L)  (0-50) | cholesterol(mol/L)  (3.55-5.20) | triglyceride(mol/L)  (0.45-1.81) | fasting blood-glucose (mol/L)  (3.89-6.11) |
| --- | --- | --- | --- | --- | --- | --- | --- | --- | --- | --- | --- | --- | --- | --- | --- | --- | --- |
| 1^6^ | female | 4 | 92 | 18.5 | UK | hepatomegaly | + | + | UK | + | _ | 503 | 180 | 279 | UK | 1.83 | 3.2 |
| 2^7^ | male | 2 | UK | UK | c.1735+1 G＞T ;  c.2546+1 G＞T  ( heterozygous) | **UK** | **UK** | **UK** | **UK** | **UK** | **UK** | **UK** | **UK** | **UK** | **UK** | **UK** | UK |
| 3^7^ | female | **2** | UK | UK | c.1735+1 G＞T  (homozygous) | **UK** | **UK** | **UK** | **UK** | **UK** | **UK** | **UK** | **UK** | **UK** | **UK** | **UK** | UK |
| 4^7^ | male | **2.5** | UK | UK | c.1735+1 G＞T ;  c.1251 dupA  ( heterozygous) | **UK** | **UK** | **UK** | **UK** | **UK** | **UK** | **UK** | **UK** | **UK** | **UK** | **UK** | UK |
| 5^7^ | male | **3** | UK | UK | c.1735+1 G＞T  (homozygous) | **UK** | **UK** | **UK** | **UK** | **UK** | **UK** | **UK** | **UK** | **UK** | **UK** | **UK** | UK |
| 6^7^ | male | **5** | UK | UK | c.4284 T＞G ;  c.202 delG  ( heterozygous) | **UK** | **UK** | **UK** | **UK** | **UK** | **UK** | **UK** | **UK** | **UK** | **UK** | **UK** | UK |
| 7^7^ | male | **7** | UK | UK | c.665-1G＞A ;  c. 4284T＞G  ( heterozygous) | **UK** | **UK** | **UK** | **UK** | **UK** | **UK** | **UK** | **UK** | **UK** | **UK** | **UK** | UK |
| 8^7^ | male | **46** | UK | UK | c.958+1G＞T ;  c. 1017delT  ( heterozygous) | **UK** | **UK** | **UK** | **UK** | **UK** | **UK** | **UK** | **UK** | **UK** | **UK** | **UK** | UK |
| 9^8^ | male | 4 | 92 | 13 | c.100C＞T ;  c. 1176-1178delTCA  ( heterozygous) | hepatomegaly | + | + | UK | + | _ | UK | 296 | 226 | 6.3 | 5.1 | 1.8 |
| 10^9^ | female | 7 | UK | UK | UK | UK | **UK** | + | UK | UK | UK | 841 | 242 | 343 | 7.0 | 4.97 | 2.0 |
| 11^9^ | male | 2 | UK | UK | UK | UK | **UK** | + | UK | UK | UK | 69 | 113 | 410 | 8.0 | 3.86 | 1.5 |
| 12^9^ | male | 4 | UK | UK | UK | UK | **UK** | + | UK | UK | splenomegaly | UK | 171 | 129 | UK | UK | 2.73 |
| 13^9^ | male | 1 | UK | UK | UK | UK | **UK** | + | UK | UK | UK | 1378 | 749 | 129 | UK | UK | 4.4 |
| 14^9^ | male | 2 | UK | UK | UK | UK | **UK** | + | UK | UK | UK | 51 | 41 | 199 | 3.09 | 4.23 | 2.9 |
| 15^9^ | male | 4 | UK | UK | UK | UK | **UK** | UK | UK | UK | UK | UK | 85 | 105 | 3.11 | 1.4 | 3.0 |
| 16^9^ | male | 12 | UK | UK | UK | UK | **UK** | UK | UK | UK | UK | UK | 164 | 152 | 5,91 | 1.24 | 5.3 |
| 17^9^ | male | 13 | UK | UK | UK | UK | **UK** | UK | UK | UK | UK | UK | 58 | 51 | 5,21 | 2.61 | 2.9 |
| 18^10^ | male | 2 | UK | UK | c.1735+1 G＞T;  c.3710-3711 delTA  ( heterozygous) | hepatomegaly | UK | UK | + | + | _ | 913 | 105 | 42 | UK | UK | 0.9 |

EMG, electromyogram ;MUP, motor unit potential; NCS ,Nerve Conduction Studies;CK, creatine kinase; ALT, alanine aminotransferase;AST, aspartate aminotransferase.
